# Invasive micropapillary carcinoma of the breast overexpresses MUC4 and is associated with poor outcome to adjuvant trastuzumab in HER2-positive breast cancer

**DOI:** 10.1186/s12885-017-3897-x

**Published:** 2017-12-28

**Authors:** María F. Mercogliano, Gloria Inurrigarro, Mara De Martino, Leandro Venturutti, Martín A. Rivas, Rosalía Cordo-Russo, Cecilia J. Proietti, Elmer A. Fernández, Isabel Frahm, Sabrina Barchuk, Daniel H. Allemand, Silvina Figurelli, Ernesto Gil Deza, Sandra Ares, Felipe G. Gercovich, Eduardo Cortese, Matías Amasino, Pablo Guzmán, Juan C. Roa, Patricia V. Elizalde, Roxana Schillaci

**Affiliations:** 1 Instituto de Biología y Medicina Experimental (IBYME-CONICET), Vuelta de Obligado 2490, C1428ADN Buenos Aires, Argentina; 2 Servicio de Patología, Sanatorio Mater Dei, C1425DND Buenos Aires, Argentina; 3 Department of Medicine, Weill Cornell Medicine, New York, NY 10021 USA; 4 UA AREA CS. AGR.ING.BIO.Y S, Universidad Católica de Córdoba, CONICET, Facultad de Ingeniería, Campus Universitario, X5016DHK Córdoba, Argentina; 5 Unidad de Patología Mamaria, Hospital General de Agudos “Juan A. Fernández”, C1425DND Buenos Aires, Argentina; 6 Servicio de Anatomía Patológica, Hospital General de Agudos “Juan A. Fernández”, C1425DND Buenos Aires, Argentina; 7 Instituto Oncológico Henry Moore, C1425DND Buenos Aires, Argentina; 8 Hospital Aeronáutico Central, C1437HPA Buenos Aires, Argentina; 9 Departamento de Anatomía Patológica (BIOREN), Universidad de La Frontera, 4811230 Temuco, Chile

**Keywords:** Invasive micropapillary carcinoma of the breast (IMPC), HER2, Mucin 4 (MUC4), Trastuzumab

## Abstract

**Background:**

Invasive micropapillary carcinoma of the breast (IMPC) is a histological tumor variant that occurs with low frequency characterized by an inside-out formation of tumor clusters with a pseudopapillary arrangement. IMPC is an aggressive tumor with poor clinical outcome. In addition, this histological subtype usually expresses human epidermal growth factor receptor 2 (HER2) which also correlates with a more aggressive tumor. In this work we studied the clinical significance of IMPC in HER2-positive breast cancer patients treated with adjuvant trastuzumab. We also analyzed mucin 4 (MUC4) expression as a novel biomarker to identify IMPC.

**Methods:**

We retrospectively studied 86 HER2-positive breast cancer patients treated with trastuzumab and chemotherapy in the adjuvant setting. We explored the association of the IMPC component with clinicopathological parameters at diagnosis and its prognostic value. We compared MUC4 expression in IMPC with respect to other histological breast cancer subtypes by immunohistochemistry.

**Results:**

IMPC, either as a pure entity or associated with invasive ductal carcinoma (IDC), was present in 18.6% of HER2-positive cases. It was positively correlated with estrogen receptor expression and tumor size and inversely correlated with patient’s age. Disease-free survival was significantly lower in patients with IMPC (hazard ratio = 2.6; 95%, confidence interval 1.1–6.1, *P* = 0.0340). MUC4, a glycoprotein associated with metastasis, was strongly expressed in all IMPC cases tested. IMPC appeared as the histological breast cancer subtype with the highest MUC4 expression compared to IDC, lobular and mucinous carcinoma.

**Conclusion:**

In HER2-positive breast cancer, the presence of IMPC should be carefully examined. As it is often not informed, because it is relatively difficult to identify or altogether overlooked, we propose MUC4 expression as a useful biomarker to highlight IMPC presence. Patients with MUC4-positive tumors with IMPC component should be more frequently monitored and/or receive additional therapies.

**Electronic supplementary material:**

The online version of this article (10.1186/s12885-017-3897-x) contains supplementary material, which is available to authorized users.

## Background

Invasive micropapillary carcinoma of the breast (IMPC) is defined as a low-frequent tumor variant of invasive carcinomas characterized by a unique inside-out formation of tumor clusters with a pseudopapillary arrangement that is present in ~6% of all breast cancers [1, 2]. These clusters are separated from each other by a clear space defined by the intervening stroma. IMPC was originally described as a histological subtype in 1980 by Fisher et al. [3], and was listed for the first time in 2003 as a histological subtype of invasive breast carcinoma in the World Health Organization (WHO) classification of breast tumors [4]. IMPC has an angioinvasive phenotype that allows its spread into blood vessels, which leads to higher rates of lymph node metastasis and poor clinical outcome [5, 6]. In addition, this histological entity is more likely to present human epidermal growth factor receptor-2 (HER2) and estrogen receptor (ER) expression [5, 7, 8]. Although IMPC constitutes a histological breast cancer subtype per se, its occurrence is most commonly associated with invasive ductal carcinoma (IDC) in which the micropapillary component is variable (mixed IMPC). Interestingly, pure IMPC harbors patterns of genomic aberrations and phenotype similar to those found in mixed IMPC [9]. In line with this evidence, it has been reported that the incidence of lymph node dissemination is independent of the relative amount of micropapillary features in the tumor. Once the micropapillary component is present in any amount, the behavior and outcome of the disease in patients with mixed IMPC are similar to those bearing pure IMPC tumors [1, 6]. In spite of this, pathologists frequently underreport this histological entity, because it is relatively difficult to identify or altogether overlooked. Therefore, it is vital to report IMPC presence, even when found in subtle proportions. A sensitive biomarker thus become instrumental in revealing its presence.

HER2-positive breast cancers are characterized by their aggressive behavior [10]. The treatment of choice is the administration of the monoclonal antibody trastuzumab associated with chemotherapy [11]. However, up to 42% of patients treated with neoadjuvant trastuzumab, and 27% of patients treated with adjuvant trastuzumab, experience disease progression [12, 13]. We have recently demonstrated that mucin 4 (MUC4) expression in HER2-positive breast cancer is a biomarker of poor prognosis in patients treated with trastuzumab in the adjuvant setting [14]. MUC4, a membrane glycoprotein, promotes metastasis given its ability to confer anti-adhesive properties to breast cancer cells [15]. In particular, we have proved that tumor necrosis factor alpha (TNFα) drives MUC4 expression in HER2-positive breast cancer through the activation of NF-kB transcription factor. We demonstrated that MUC4 induced by TNFα is able to shield trastuzumab epitope on the HER2 molecule and constitutes a mechanism by which TNFα promotes trastuzumab resistance in HER2-positive cancers [14]. Interestingly, TNFα presence has been associated with microvessel density in IMPC [16]. Since pathologists’ examination of our HER2-positive cohort revealed several IMPC cases, and because MUC4 and IMPC share several characteristics [15, 16], we wanted to explore whether MUC4 expression could be a feature of this breast cancer subtype.

To our knowledge, the IMPC data published so far focused on comparing this histological breast cancer subtype with others. Here, we present a study of IMPC incidence in HER2-positive breast cancer patients and its potential clinical significance on responsiveness benefit to adjuvant trastuzumab and chemotherapy. We also evaluated whether the expression of MUC4 by IHC could be a useful biomarker of the IMPC histological type.

## Methods

### Patients

Breast cancer paraffin-embedded tissue sections of 86 consecutive patients with HER2-postive primary breast cancer were retrieved from the Pathology Department of Hospital Juan A. Fernández, Instituto de Oncología Henry Moore, (Buenos Aires, Argentina) and Hospital de Temuco, (Temuco, Chile) from 2005 to 2014. The median follow-up time was 30 months (range 0.5–9 years). Also, a cohort of 113 consecutive breast cancer samples from Instituto de Oncología Henry Moore was included in the analysis as a control. This study was conducted under the provisions of the Declaration of Helsinki and informed written consents were obtained from all patients before inclusion. Study protocols were approved by the Ethic Committees of the participating institutions. Patients were included if they had received adjuvant trastuzumab and chemotherapy treatment, had complete data on baseline clinical features and treatment outcomes, and were preoperatively chemotherapy and radiotherapy *naïve*. Patients received standard adjuvant chemotherapy plus 1 year of treatment with trastuzumab: 4 cycles, one every three weeks, of doxorubicin (60 mg/m^2^ i.v.) plus cyclophosphamide (600 mg/m^2^ i.v.) followed by 4 cycles every 3 weeks of paclitaxel (80 mg/m^2^ i.v.), plus trastuzumab (8 mg/kg i.v. loading dose with first dose of docetaxel followed by 6 mg/kg every 3 weeks for 1 year). Pre-treatment patient staging was classified according to the American Joint Committee on Cancer (AJCC) system through the Elston and Ellis histological grading system. Tumor specimens were anonymized for this study.

### Histopathological analysis and immunohistochemistry (IHC)

For IHC, antigen retrieval was performed in 5 μm paraffin embedded tissue sections in positively charged slides in 10 mmol/L sodium citrate buffer pH 6 for 50 min at 92 °C. Slides were incubated with antibodies against MUC4 (1:50, 1G8 Santa Cruz Biotechnology, Santa Cruz, CA, 1:50) or TNFα (#9739, 1:100, Abcam, Cambridge, United Kingdom) overnight at 4 °C. Sections were then incubated with a biotinilated anti-mouse or biotinilated anti-rabbit secondary antibody, dilution 1:400, for 30 min and then with incubated with the VECTASTAIN® Elite® ABC-HRP Kit (Vector, Burlingame, CA) and developed with DAB (3,3′-diaminobenzidine) (Cell Marque, Rocklin, CA). MUC4 quantification was done using a score of 0 to 3+, as previously reported by Workman et al. [17]. TNFα quantification was done as we previously reported [14]. The scoring system was the following: score: 0, no stain to less than 30% of cells staining faintly; 1+, over 30% of cells staining light to moderate; 2+, over 50% of cells staining moderately; 3+, intense staining of majority of the epithelial population [14]. Immunostainings were run with known positive and negative tissue controls. Expression and localization of the proteins were independently evaluated by three pathologists, GI, IF and MA, who were masked to clinical data and treatment outcome. Score discrepancies were re-evaluated and reconciled on a multiple-headed microscope. Tumors were considered MUC4-positive or TNFα positive when they exhibited a score of 2+ or 3+ [14]. HER2 was evaluated by IHC with the polyclonal antibody A0485 (Dako) and was scored according to the American Society of Clinical Oncology/College of American Pathologists guidelines (ASCO/CAP). Tumors were considered HER2-positive if they presented a score of 3+ by IHC or score 2+ and confirmed HER2 amplification by FISH (PathVysion™, Vysis Inc., Downers Grove, IL). The immunohistochemical assessment of ER and PR receptors was made using the 6F11 (Novocastra Laboratories, U.K) and 1A6h PRa2 + hPRa3 (NeoMarkers, Freemont, CA) antibodies, respectively, and were scored as described previously [18]. Guidelines for Reporting Recommendations for Tumor Marker Prognostic Studies (REMARK) were followed in this work [19].

### MUC4 validation cohort

In order to evaluate MUC4 in an independent patient cohort, we used gene expression data from 113 patients. Data is available from ArrayExpress® repository under accession number “E-NCMF-3”. The data set was evaluated and processed (quantile normalized) by means of the lmdme [20] and the limma [21] libraries from Bioconductor® repository and run into the R environment [22]. Differential gene expression was analyzed through the “eBayes” function from limma. The script code is available in the Additional file 1.

### Statistical analysis

Analyses were performed using SPSS software version 15.0 (SPSS Inc.; Chicago, IL). Correlations between categorical variables were performed using the χ2-test or Fisher’s exact test when the number of observations obtained for analysis was under five. Disease-free survival (DFS) was calculated from the date of initial diagnosis to the date of recurrence or death, whichever came first. Cumulative DFS probabilities were calculated according to the Kaplan-Meier method and statistical significance was analyzed by log-rank test or Wilcoxon test. For univariate analysis, we used the Cox proportional hazards regression model. The hazard ratio (HR) and its 95% confidence interval (CI) were calculated for each variable. Statistical differences of MUC4 expression were determined by Kruskal-Wallis test and Dunn’s test using GraphPad Prism 6 software (GraphPad Software, La Jolla, CA, USA). *P* values under 0.05 were considered statistically significant and all reported *P* values were 2-sided.

## Results

### IMPC is associated with poor outcome in HER2-positive breast cancer patients

**Fig. 1:**
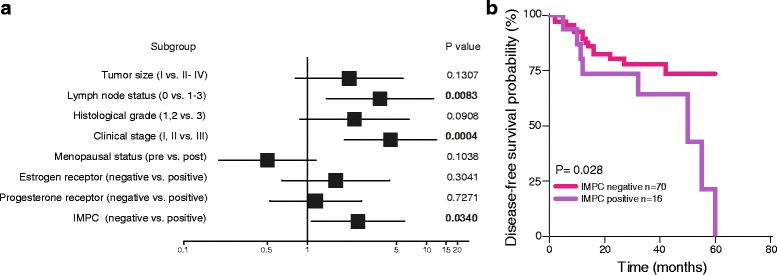
IMPC is associated with poor outcome to adjuvant trastuzumab treatment in HER2-positive breast cancer patients. **a** Forest plot showing the hazard ratios (HR, squares) and 95% confidence intervals (CI, horizontal lines) of Cox univariate subgroup analysis. **b** Kaplan–Meier analysis of the probability of DFS of patients who received adjuvant trastuzumab treatment, based on the presence of IMPC. Log rank test was used

As HER-2 expression is frequently observed in IMPC [8], our purpose was to study the incidence and clinical relevance of this histological breast cancer subtype in HER-2 positive breast cancer patients. We have a cohort of 86 HER2-positive breast cancer patients, treated with trastuzumab and chemotherapy (see details in Methods) in the adjuvant setting, whose clinicopathological features are shown in Table 1. We found that 16 tumors (18.6% of the HER-2-positive tumors) were either pure (6 cases) or had different proportions of IMPC component mixed with IDC (IMPC 10–30%:4 cases; 31–70%: 2 cases; 71–90%: 4 cases). In accordance with previous reports [23], we observed that IMPC presence (pure and mixed cases) was associated with younger patients, larger tumor size and positive ER expression, which we considered hormone receptor (HR) positive (Table 2). We observed a trend of association between IMPC and lymph node status (Table 2). Interestingly, univariate analysis showed that IMPC was associated with poor DFS (HR = 2.6; 95% CI 1.1–6.1; *P* = 0.0340) (Fig. 1a). Also, lymph node metastasis (HR = 3.8; 95%, CI: 1.4–10.5; *P* = 0.0083) and clinical stage status (HR = 4.7; 95%CI 2.0–11.0; *P* = 0.00004) was associated with reduced DFS (Fig. 1a). Kaplan-Meier analysis revealed that IMPC presence was associated with reduced DFS in patients treated with standard trastuzumab treatment in the adjuvant setting (log rank *P* = 0.028; Fig. 1b). No differences were observed in DFS between patients with pure (*n* = 6) and mixed IMPC (*n* = 10, *P* = 0.594, data not shown). Our results show that the presence of IMPC, either as a pure or mixed entity, reveals a subgroup of HER2-positive breast cancer patients with poor outcome to adjuvant trastuzumab and chemotherapy.

**Table 1:** Clinicopathological characteristics of the HER2+ cohort

Characteristic	*N*° patients	%	Median	Range
Total number of patients	86			
Age (years)			50	25–79
Length follow-up (months)			30	6–112
Menopausal status				
Pre	48	55.8		
Post	38	44.2		
Tumor size				
T1	33	38.8		
T2	36	42.4		
T3	12	14.1		
T4	4	4.7		
Not documented	1			
Lymph node status				
N0	44	51.2		
N1	30	34.9		
N2	5	5.8		
N3	7	8.1		
Clinical stage				
I	25	29.1		
II	40	46.5		
III	21	24.4		
Histological grade				
1	7	8.9		
2	28	35.4		
3	44	55.7		
Not documented	7			
Estrogen receptor positive	66.3			
Progesterone receptor positive	55.8

**Table 2:** Association between IMPC and clinicopathological characteristics

Clinicopathological characteristics		non-IMPC	IMPC	*P*
		*n* (%)	*n* (%)	
Menopausal status	pre	35 (50.0)	13 (81.3)	*0.021*
	post	35 (50.0)	3 (18.8)	
Tumor size	1	31 (44.9)	2 (12.5)	*0.014*
	2–4	38 (55.1)	14 (87.5)	
Lymph node status	0	39 (55.7)	5 (31.3)	0.068
	1–3	31 (44.3)	11 (68.7)	
Clinical stage	I, II	52 (74.3)	13 (81.3)	0.41
	III	18 (25.7)	3 (18.7)	
Histological grade	1,2	30 (46.2)	5 (35.7)	0.447
	3	35 (53.8)	9 (64.3)	
Estrogen receptor	Negative	27 (38.6)	2 (12.5)	*0.04*
	Positive	43 (61.4)	14 (87.5)	
Progesterone receptor	Negative	33 (47.1)	5 (31.3)	0.248
	Positive	37 (52.9)	11 (68.7)	

The numbers in italic correspond to statistically significant *p* values

### MUC4 is overexpressed in IMPC

**Fig. 2:**
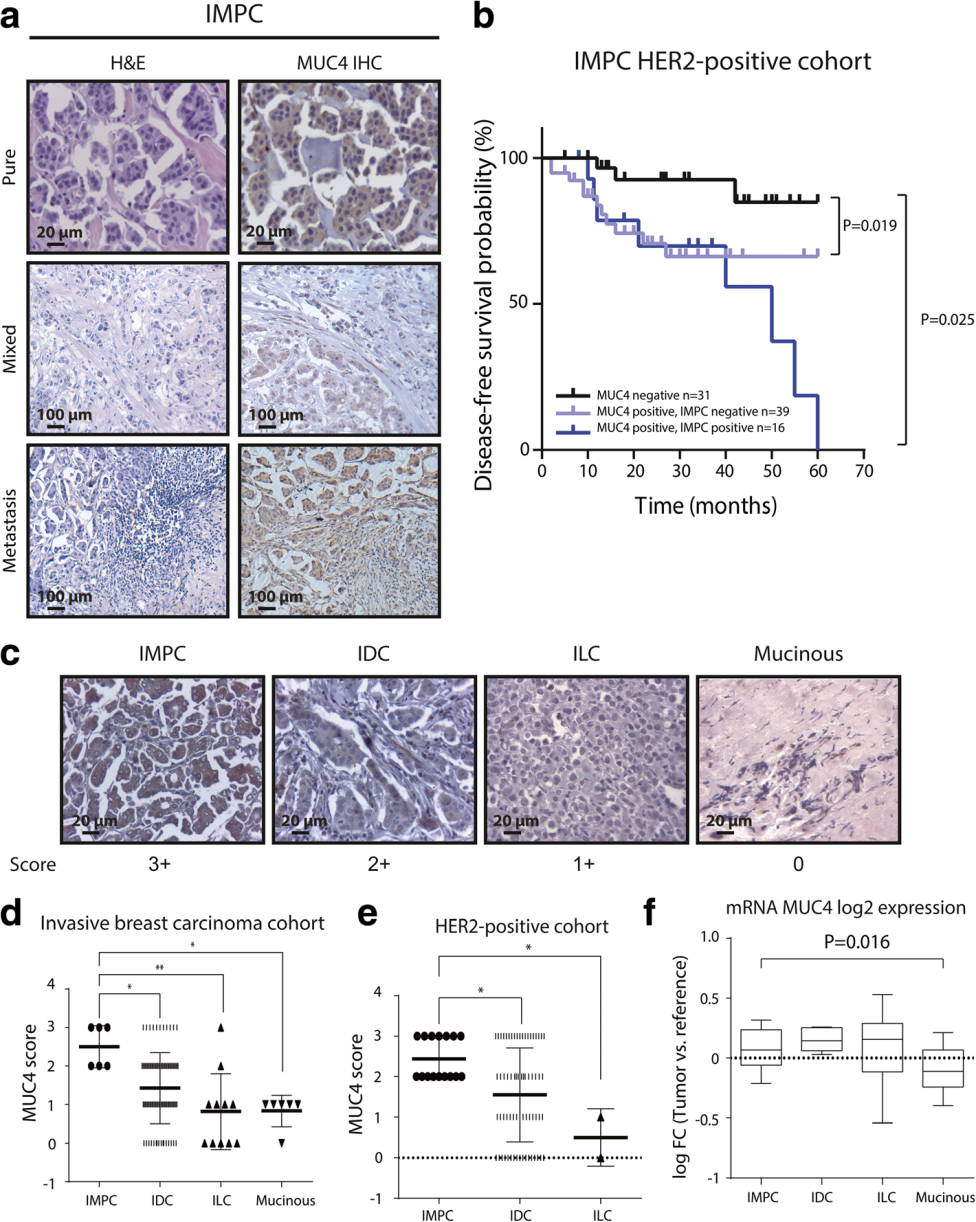
MUC4 is overexpressed in IMPC. **a** Representative images of H&E and MUC4 staining by IHC of pure, mixed and metastatic IMPC. **b** Kaplan–Meier analysis of the probability of DFS of patients who received adjuvant trastuzumab treatment, based on the expression of MUC4 and IMPC. **c** Representative images of MUC4 staining of IMPC in different histological breast cancer subtypes by IHC. MUC4 expression was scored according to Workman et al. [17]. **d** and **e** Scores of MUC4 expression classified in the histological subtypes IMPC, IDC, ILC and mucinous carcinoma in a cohort of 113 invasive breast cancer samples and in the 86 HER2-positive breast cancer cohort respectively. **f** Log Fold Change (FC) expression (Tumor vs. Universal reference) sample distribution for MUC4 over each breast cancer subtype. *P < 0.05, **P < 0.01

We already demonstrated that MUC4 expression is a biomarker of resistance to adjuvant trastuzumab treatment [14]. As we found that 18.6% of the HER2-positive cohort analyzed had IMPC differentiation (pure or mixed) and showed poor outcome to trastuzumab and chemotherapy, we explored whether MUC4 is expressed in IMPC cases. MUC4 detection by IHC showed strong cytoplasmic staining in all IMPC tested (Table 3 *, P* = 0.0003). This was also true for 20 additional cases of IMPC, whose follow-up data was not available, and was not included in this study. MUC4 positivity was observed in IMPC located in nodal metastasis and in primary tumors, independently of the percentage of IMPC present in the sample (Fig. 2a).

**Table 3:** Association between MUC4 expression and IMPC

MUC4	non-IMPC	IMPC	*P*
	*n* (%)	*n* (%)	
Negative	31 (44.3)	0 (0)	*0.0003*
Positive	39 (55.7)	16 (100)	

The numbers in italic correspond to statistically significant *p* values

As we observed that a subgroup of MUC4-positive tumors had IMPC differentiation, we compared the DFS of patients whose tumors were MUC4-negative, MUC4-positive without IMPC and MUC4-positive with IMPC component. Figure 2b shows that MUC4-negative patients benefit more from trastuzumab than MUC4-positive patients (either with or without IMPC). However, MUC4-positive patients with IMPC tumors tended to have lower DFS after 3 years of the onset of treatment than the ones having MUC4-positive tumors without IMPC. In addition, patients with tumors MUC4-positive and IMPC (pure or mixed) were younger than patients with MUC4-positive without IMPC and MUC4-negative tumors (Table 4).

**Table 4:** Association of expression of MUC4 and IMPC with age at diagnosis

	Expression of MUC4 and IMPC			
Age (years)	MUC4 negative	MUC4 positive/IMPC negative	MUC4 positive/IMPC positive	*P*
< 50	13 (42.0)	22 (56.4)	13 (81.3)	*0.036*
≥50	18 (58.0)	17 (43.6)	3 (18.7)	

The numbers in italic correspond to statistically significant *p* values

To compare MUC4 expression in IMPC with respect to other histological breast cancer subtypes, we performed IHC staining on an independent cohort of 113 breast cancer samples used as control. Their baseline clinicopathological data is shown in Additional file 2: Table S1. Histological analysis of this cohort showed that 79.6% were IDC, 9,8% infiltrating lobular carcinoma (ILC), 5.3% mucinous carcinoma and 5.3% IMPC (Additional file 3: Table S2).We observed that IMPC is the histological entity with the highest MUC4 expression (Fig. 2c and d). Contrastingly, infiltrating lobular carcinoma (ILC) and mucinous carcinoma expressed MUC4 faintly, while IDC exhibited intermediate expression levels (Fig. 2c and d). Concordantly, in our cohort of 86 HER2-positive patients, MUC4 score in IMPC was higher than in IDC (Fig. 2e).

To validate our findings, data from Lopez-Garcia et al. [24] was used to contrast the gene expression of MUC4 mRNA (ENST00000314335) levels among IMPC, IDC, ILC and mucinous carcinoma using linear models of microarray data (Limma) [21]. In spite of the small number of cases reported in this study (IMPC, *n* = 8, IDC, *n* = 10, ILC, *n* = 20; mucinous carcinoma, n = 10), the data showed a statistically significant increase in mRNA MUC4 levels in IMPC with respect to those in mucinous carcinoma (Fig. 2f *, P* = 0.0164). mRNA MUC4 levels in IMPC did not show significant differences between IDC and ILC in this data set. Interestingly, the differences of MUC4 protein expression in IDC and ILC were not seen at mRNA level. All these results proved that MUC4 is overexpressed in IMPC and that it is a sensitive biomarker useful to show IMPC presence.

## Discussion

Here we found that 18.6% of HER-2 positive breast cancers have IMPC differentiation (pure or mixed entities) in contrast to the 6% reported in breast cancer in general statistics [1, 2]. This characteristic was more strongly associated with HER-2 positive/HR positive tumors (14/57, 24.6%) than with HER2-positve/HR-negative breast cancer subtype (2/29, 6.9%). To our knowledge, this is the first study in which IMPC incidence is explored in a HER2 positive cohort. It has been described that between 30 and 80% of IMPC are HER2-positive [7, 8]. However, the impact of IMPC on trastuzumab efficacy has not been explored. Here, we revealed that IMPC is associated with poor DFS of patients treated with adjuvant trastuzumab and chemotherapy.

Interestingly, our results clearly show that MUC4 is overexpressed in IMPC as compared to IDC, ILC and mucinous carcinoma. Cytoplasmic staining of MUC4 is strong in all the studied IMPC cases including those with metastatic lesions and primary tumors, even when the IMPC component is as small as 10%. These results based on MUC4 protein expression by IHC were also confirmed in silico, using mRNA from an independent cohort [24] where a significant increase in MUC4 mRNA levels in IMPC was found with respect to the mucinous carcinoma. MUC4 mRNA levels could attained no statistical significance when comparing IMPC with IDC or ILC.. Other biomarkers have been used to characterize IMPC. For example, mucin 1 (MUC1) is present at the reversed apical membrane of IMPC clusters. However, MUC1 exhibits a membrane pattern together with cytoplasmic staining in the mixed IMPC, rendering the membrane staining only a marker of the pure entity [25]. The epithelial membrane antigen (EMA) also shows the “inside-out” staining in IMPC, but IDCs with osteoclastic giant cells also have a similar EMA staining pattern [26]. Recently, it was reported that p120 immunostaining was useful to determine the presence of IMPC [27]. In fact, p120 has the advantage of showing membrane staining with a cleaner background than that of EMA IHC, resulting in a better determination of IMPC features. Our previous report demonstrated that MUC4, induced by TNFɑ, shields the trastuzumab binding epitope on the HER2 molecule and therefore antibody dependent cell cytotoxicity is impaired [14]. In addition, TNFα presence has been positively associated with microvessel density (MVD) in IMPC [16]. We also observed strong intensity of TNFα staining in all IMPC samples (Additional file 4: Figure S1 data not shown). It would be useful to have a biomarker panel to determine IMPC by quantification of MVD, and VEGF, p120 and MUC4 staining by IHC. We also proved that MUC4 expression is associated with resistance to adjuvant trastuzumab administration and chemotherapy in HER2-positive breast cancer patients. Taken together, our results show that, MUC4 presents itself as a sensitive biomarker for IMPC detection. In addition, we postulate that the expression of MUC4 in IMPC could be one of the causes of the aggressive behavior of this tumor.

Several reports have acknowledged that IMPC is associated with worse prognosis [28, 29]. In line with this evidence, our work showed that IMPC in HER2-positive breast cancer is associated with ER expression, younger patients and poor benefit to standard adjuvant trastuzumab and chemotherapy. In conclusion, our findings strongly recommend seeking out the IMPC component, and informing its presence, even if the IMPC component is subtle, particularly in the case of HER2 positive/HR positive breast cancer. We therefore suggest the introduction of MUC4 determination to help identify the IMPC component. There are no specific treatments today for IMPC, but pursuant to our findings, the oncologist should subject patients to more frequent monitoring and other HER2-targeted therapies (i.e.pertuzumab) and/or TNFα blocking strategies.

## Conclusion

In this work we have shown that MUC4 is overexpressed in IMPC and, moreover, MUC4 staining by IHC is a useful biomarker to define IMPC presence, a difficult task since IMPC tends to be overlooked and there are no reliable biomarkers available. We propose a panel of biomarkers to determine the micropapillary histological subtype consisting of MUC4, VEGF y p120. We also showed that IMPC in HER2-positive breast cancer is associated with ER expression, younger patients and poor benefit to standard adjuvant trastuzumab and chemotherapy. In conclusion, our findings strongly recommend seeking and informing IMPC presence, even if the micropapillary component is subtle, particularly in the case of HER2 positive/HR positive breast cancer. Since there are not specific treatments available today for IMPC, the results exposed in this work indicate that the oncologist should subject patients to more frequent monitoring and other HER2-targeted treatments (i.e pertuzumab) and/or TNFα blocking strategies to provide a better outcome for HER2 positive/HR positive IMPC breast cancer patients.

## Additional files

Additional file 1 Supplementary data. (DOC 50 kb)

Additional file 2: Table S1. Clincopathological characteristics of the cohort. (XLS 30 kb)

Additional file 3: Table S2. Histological subtypes of the cohort (*n* = 113). (XLSX 9 kb)

Additional file 4: Figure S1. TNFα staining in IDC and IMPC by immunohistochemistry. The panels show representative cases of IDC and IMPC for H&E staining (upper panel), MUC4 (middle panel) and TNFα (lower panel). (TIFF 5225 kb)

## Abbreviations

CI: Confidence interval
DFS: Disease-free survival
ER: Estrogen receptor
HR: Hazard ratio
HR+: Hormone receptor positive
IDC: Invasive ductal carcinoma
IHC: Immunohistochemistry
ILC: Infiltrating lobular carcinoma
IMPC: Invasive micropapillary carcinoma of the breast
MUC4: Mucin 4
MVD: Microvessel density
PR: Progesterone receptor
TNFα: Tumor necrosis factor alpha
