# Supplementary material for: Invasive micropapillary carcinoma of the breast overexpresses MUC4 and is associated with poor outcome to adjuvant trastuzumab in HER2-positive breast cancer
Source: BMC Cancer. 2017 Dec 28;17:895. doi: 10.1186/s12885-017-3897-x (PMC5745882; doi:10.1186/s12885-017-3897-x)
Supplement: Supplementary file 1 — Supplementary data. (DOC 50 kb) [file 12885_2017_3897_MOESM1_ESM.doc]

**Supplementary data**

This file present the R script used to analyze the Validation Cohort

**#script R**

**## the data has been downloaded from http://www.ebi.ac.uk/arrayexpress/files/E-NCMF-3/E-NCMF-3.eSet.r**

**# file:~/E-MTAB-365.eSet.r**

**load("~/E-NCMF-3.eSet.r")**

**library(Biobase)**

**library(ArrayExpress)**

**pd <- pData(study)**

**fd <- fData(study)**

**##using limma**

**library(limma)**

**library(lmdme)**

**##building the RGList object**

**RG.data <- new( "RGList",list( R = assayData(study)[["R"]],**

**G = assayData(study)[["G"]],**

**Rb = assayData(study)[["Rb"]],**

**Gb = assayData(study)[["Gb"]] ))**

**rm(study)**

**MA.data <- MA.RG(RG.data)**

**##editing the information for each DYE chanel**

**cy3 <- unlist(lapply(pd$Source.Name.Cy3, function(x) unlist(strsplit(x,"_"))[1]))**

**cy5 <- unlist(lapply(pd$Source.Name.Cy5, function(x) unlist(strsplit(x,"_"))[1]))**

**#editing the names of each breast cancer type**

**acortar <- function(cy){**

**cy[cy=="AdenoidCystic"] <- "Ad"**

**cy[cy=="Apocrine"] <- "Ap"**

**cy[cy=="Endocrine"] <- "En"**

**cy[cy=="Medullary"] <- "Md"**

**cy[cy=="Metaplastic"] <- "Me"**

**cy[cy=="Micropapillary"] <- "Mi"**

**cy[cy=="MucinousA"] <- "MA"**

**cy[cy=="MucinousB"] <- "MB"**

**cy[cy=="Tubular"] <- "Tu"**

**return(cy)**

**}**

**cy3 <- acortar(as.character(cy3))**

**cy5 <- acortar(as.character(cy5))**

**##Normalize the data of all subjects**

**MA.qn <- normalizeBetweenArrays(MA.data,method="quantile")**

**##setting column names**

**colnames(MA.qn$M) <- cy3**

**colnames(MA.qn$M)[cy3 == "Reference Pool"] <- cy5[ cy5 != "Reference Pool"]**

**##build the target data frame for experiment design information**

**cn <- cbind(pd$Source.Name.Cy5, pd$Source.Name.Cy3)**

**cn2 <- cn[,1]**

**cn2[cn2 == "Reference Pool" ] <- cn[cn2 == "Reference Pool",2]**

**cn <- cbind(pd$Source.Name.Cy5, pd$Source.Name.Cy3,cn2)**

**colnames(cn) <- c("Cy5", "Cy3", "Subj")**

**Trat <- unlist(lapply(cn[,3], function(x) unlist(strsplit(x, "_"))[1]))**

**Sj <- unlist( lapply(cn[,3], function(x) {**

**xx <- unlist(strsplit(x, "_"))**

**if(length(xx)>2) return(xx[3])**

**return(xx[2])**

**} ) )**

**cn <- cbind(cn, Trat=Trat, Sjn = Sj)**

**cn <- cbind(cn, Treatment=acortar(cn[, "Trat"]))**

**dim(table(cn[,3]))**

**# [1] 113**

**##assign the target**

**MA.qn$targets <- as.data.frame(cn)**

**MA.qn$targets$Treatment <- as.factor(MA.qn$targets$Treatment)**

**MA.qn$targets$Subject <- as.factor(MA.qn$targets$Sjn)**

**MA.qn$targets$Dye <- "Cy5"**

**MA.qn$targets$Dye[MA.qn$targets$Cy5 == "Reference Pool"] <- "Cy3"**

**MA.qn$targets$Dye <- as.factor(MA.qn$targets$Dye)**

**table(MA.qn$targets$Dye)**

**##assign gene information**

**MA.qn$genes <- fd**

**# source file:~/human_V3.0.2_genelist_s-_all OMAD data.xls**

**##Some interesting genes to explore, only those found in ENSEMBL**

**#identifying MUC4**

**MUC4 <- c("ENST00000314335","ENST00000314268")##the first transcript was used.**

**MUC4.id <-which(MA.qn$genes$Reporter.Database.Entry.ens_trscrpt_id. %in% MUC4)**

**########flip the Cy3 values**

**Mnew <- MA.qn**

**Mnew$M[, Mnew$targets$Dye == "Cy3"] <- -Mnew$M[, Mnew$targets$Dye == "Cy3"]**

**##control**

**##we use lmdme to verify Dye effects**

**fit.lmdme <- lmdme(model = ~Trat*Dye, data = na.omit(M), design = target.total, Bayes = TRUE, verbose = TRUE)**

**decomposition(fit.lmdme, "pca", scale = "row", type = "residual")**

**biplot(fit.lmdme, term = "Dye", xlabs = ".", expand = 0.9) ## strong Dye bias effect. So, it should be**

**#taken into account in the differential gene expression model.**

**##model using MA as the mean reference**

**Mnew.BCinterest <- Mnew[, Mnew$targets$Treatment %in% c("MA","Mi","IDC") ]**

**Mnew.BCinterest$targets$Treatment <- factor(as.character(Mnew.BCinterest$targets$Treatment), labels = c("MA","Mi","IDC"))**

**##model using Mi as the mean reference**

**Mnew.BCinterest$targets$Treatment <- factor(as.character(Mnew.BCinterest$targets$Treatment), labels = c("Mi","MA","IDC"))**

**Mnew.BCinterest$targets$Subject <- factor(as.character(Mnew.BCinterest$targets$Subject))**

**design <- model.matrix(~Treatment+Dye, Mnew.BCinterest$targets)**

**block <- Mnew.BCinterest$targets$Subject**

**table(block)**

**dupcor <- duplicateCorrelation(Mnew.BCinterest$M ,design,block=block)**

**dupcor$consensus.correlation**

**fit3 <- eBayes(lmFit(Mnew.BCinterest$M,design,block=block,correlation=dupcor$consensus))**

**round(fit3$p.value[MUC4.id[1],],4)**

**round(fit3$coefficients[MUC4.id[1],],4)**

**#MA as reference**

**# (Intercept) TreatmentMi TreatmentIDC DyeCy5**

**# 0.0850 0.0166 0.5770 0.0000**

**#coefficients (Log Fold Change)**

**# (Intercept) TreatmentMi TreatmentIDC DyeCy5**

**# 0.1428 -0.2408 -0.0565 -0.1660**

**#Pvalues Mi as reference**

**# (Intercept) TreatmentMA TreatmentIDC DyeCy5**

**# 0.1916 0.5770 0.0330 0.0000**

**round(fit3$coefficients[MUC4.id[1],],4)**

**#coefficients (Log Fold Change)**

**# (Intercept) TreatmentMA TreatmentIDC DyeCy5**

**# 0.0863 0.0565 -0.1842 -0.1660**

**##analyzing only MUC4 expression levels over the MA, Mi and IDC**

**library(ggplot2)**

**data2 <-data.frame(MUC4=Mnew$M[MUC4.id[1],],Type=Mnew$targets$Treatment:Mnew$targets$Dye, Treatment = Mnew$targets$Treatment,**

**Dye = Mnew$targets$Dye, Subject = Mnew$targets$Subject )**

**ggplot(data2, aes(Type, MUC4 )) + geom_boxplot(aes(color = Treatment)) + geom_jitter()**

**summary(lm(MUC4~Treatment*Dye+Subject, data = data2))**

**data3 <- subset(data2, Treatment %in% c("MA","Mi","IDC"))**

**data3$Treatment <- factor(as.character(data3$Treatment), labels = c("Mi","MA","IDC"))**

**data3$Subject <- as.factor(as.character(data3$Subject))**

**summary(lm(MUC4 ~ Treatment*Dye+Subject,data3))**

**#**

**# Call:**

**# lm(formula = MUC4 ~ Treatment * Dye + Subject, data = data3)**

**#**

**# Residuals:**

**# Min 1Q Median 3Q Max**

**# -0.21352 -0.05535 0.00000 0.05535 0.21352**

**#**

**# Coefficients: (2 not defined because of singularities)**

**# Estimate Std. Error t value Pr(>|t|)**

**# (Intercept) 0.15091 0.09072 1.663 0.111808**

**# TreatmentMA -0.52787 0.12560 -4.203 0.000438 *****

**# TreatmentIDC -0.19750 0.12628 -1.564 0.133496**

**# DyeCy5 -0.19103 0.07407 -2.579 0.017926 ***

**# Subject3671 0.33839 0.11712 2.889 0.009069 ****

**# Subject3672 0.13945 0.11712 1.191 0.247711**

**# Subject3673 0.16448 0.11712 1.404 0.175544**

**# Subject3674 0.09080 0.11712 0.775 0.447238**

**# Subject3675 0.13084 0.11712 1.117 0.277179**

**# Subject3676 0.37535 0.11712 3.205 0.004447 ****

**# Subject3677 0.60520 0.11712 5.167 4.68e-05 *****

**# Subject3678 0.70163 0.11712 5.991 7.39e-06 *****

**# Subject3679 0.29210 0.11712 2.494 0.021507 ***

**# Subject3680 NA NA NA NA**

**# Subject3726 0.04806 0.11712 0.410 0.685905**

**# Subject3727 -0.05718 0.11712 -0.488 0.630718**

**# Subject3728 0.05144 0.11712 0.439 0.665203**

**# Subject3729 0.13646 0.11712 1.165 0.257665**

**# Subject3730 -0.11353 0.11712 -0.969 0.343931**

**# Subject3731 0.30798 0.11712 2.630 0.016064 ***

**# Subject3732 0.31090 0.11712 2.655 0.015215 ***

**# Subject3734 0.28235 0.11712 2.411 0.025659 ***

**# Subject3735 0.15797 0.11712 1.349 0.192468**

**# Subject3736 -0.10873 0.11712 -0.928 0.364305**

**# Subject3738 -0.04185 0.11712 -0.357 0.724600**

**# Subject3739 NA NA NA NA**

**# TreatmentMA:DyeCy5 0.01533 0.09072 0.169 0.867485**

**# TreatmentIDC:DyeCy5 0.05278 0.09443 0.559 0.582425**

**# ---**

**# Signif. codes: 0 ‘***’ 0.001 ‘**’ 0.01 ‘*’ 0.05 ‘.’ 0.1 ‘ ’ 1**

**#**

**# Residual standard error: 0.1171 on 20 degrees of freedom**

**# Multiple R-squared: 0.8902, Adjusted R-squared: 0.753**

**# F-statistic: 6.488 on 25 and 20 DF, p-value: 3.663e-05**

**summary(lm(MUC4 ~ 0+Treatment*Dye+Subject,data3))**

**#**

**# Call:**

**# lm(formula = MUC4 ~ 0 + Treatment * Dye + Subject, data = data3)**

**#**

**# Residuals:**

**# Min 1Q Median 3Q Max**

**# -0.21352 -0.05535 0.00000 0.05535 0.21352**

**#**

**# Coefficients: (2 not defined because of singularities)**

**# Estimate Std. Error t value Pr(>|t|)**

**# TreatmentMi 0.15091 0.09072 1.663 0.111808**

**# TreatmentMA -0.37696 0.08686 -4.340 0.000318 *****

**# TreatmentIDC -0.04659 0.08784 -0.530 0.601677**

**# DyeCy5 -0.19103 0.07407 -2.579 0.017926 ***

**# Subject3671 0.33839 0.11712 2.889 0.009069 ****

**# Subject3672 0.13945 0.11712 1.191 0.247711**

**# Subject3673 0.16448 0.11712 1.404 0.175544**

**# Subject3674 0.09080 0.11712 0.775 0.447238**

**# Subject3675 0.13084 0.11712 1.117 0.277179**

**# Subject3676 0.37535 0.11712 3.205 0.004447 ****

**# Subject3677 0.60520 0.11712 5.167 4.68e-05 *****

**# Subject3678 0.70163 0.11712 5.991 7.39e-06 *****

**# Subject3679 0.29210 0.11712 2.494 0.021507 ***

**# Subject3680 NA NA NA NA**

**# Subject3726 0.04806 0.11712 0.410 0.685905**

**# Subject3727 -0.05718 0.11712 -0.488 0.630718**

**# Subject3728 0.05144 0.11712 0.439 0.665203**

**# Subject3729 0.13646 0.11712 1.165 0.257665**

**# Subject3730 -0.11353 0.11712 -0.969 0.343931**

**# Subject3731 0.30798 0.11712 2.630 0.016064 ***

**# Subject3732 0.31090 0.11712 2.655 0.015215 ***

**# Subject3734 0.28235 0.11712 2.411 0.025659 ***

**# Subject3735 0.15797 0.11712 1.349 0.192468**

**# Subject3736 -0.10873 0.11712 -0.928 0.364305**

**# Subject3738 -0.04185 0.11712 -0.357 0.724600**

**# Subject3739 NA NA NA NA**

**# TreatmentMA:DyeCy5 0.01533 0.09072 0.169 0.867485**

**# TreatmentIDC:DyeCy5 0.05278 0.09443 0.559 0.582425**

**# ---**

**# Signif. codes: 0 ‘***’ 0.001 ‘**’ 0.01 ‘*’ 0.05 ‘.’ 0.1 ‘ ’ 1**

**#**

**# Residual standard error: 0.1171 on 20 degrees of freedom**

**# Multiple R-squared: 0.898, Adjusted R-squared: 0.7655**

**# F-statistic: 6.776 on 26 and 20 DF, p-value: 2.457e-05**

**> sessionInfo()**

**R version 3.3.1 (2016-06-21)**

**Platform: x86_64-pc-linux-gnu (64-bit)**

**Running under: Ubuntu 16.04.1 LTS**

**locale:**

**[1] LC_CTYPE=en_US.UTF-8 LC_NUMERIC=C LC_TIME=es_AR.UTF-8 LC_COLLATE=en_US.UTF-8**

**[5] LC_MONETARY=es_AR.UTF-8 LC_MESSAGES=en_US.UTF-8 LC_PAPER=es_AR.UTF-8 LC_NAME=C**

**[9] LC_ADDRESS=C LC_TELEPHONE=C LC_MEASUREMENT=es_AR.UTF-8 LC_IDENTIFICATION=C**

**attached base packages:**

**[1] parallel stats graphics grDevices utils datasets methods base**

**other attached packages:**

**[1] statmod_1.4.26 ggplot2_2.1.0 lmdme_1.14.0 stemHypoxia_1.8.0 pls_2.5-0**

**[6] ArrayExpress_1.32.0 Biobase_2.32.0 BiocGenerics_0.18.0 limma_3.28.21**

**loaded via a namespace (and not attached):**

**[1] Rcpp_0.12.7 BiocInstaller_1.22.3 GenomeInfoDb_1.8.7 plyr_1.8.4**

**[5] XVector_0.12.1 iterators_1.0.8 tools_3.3.1 zlibbioc_1.18.0**

**[9] digest_0.6.10 bit_1.1-12 RSQLite_1.0.0 preprocessCore_1.34.0**

**[13] gtable_0.2.0 ff_2.2-13 foreach_1.4.3 DBI_0.5-1**

**[17] affxparser_1.44.0 Biostrings_2.40.2 S4Vectors_0.10.3 IRanges_2.6.1**

**[21] stats4_3.3.1 grid_3.3.1 XML_3.98-1.4 oligo_1.36.1**

**[25] scales_0.4.0 codetools_0.2-14 oligoClasses_1.34.0 GenomicRanges_1.24.3**

**[29] splines_3.3.1 SummarizedExperiment_1.2.3 colorspace_1.2-6 labeling_0.3**

**[33] munsell_0.4.3 affyio_1.42.0**
