# Supplementary figures and images for: Invasive micropapillary carcinoma of the breast overexpresses MUC4 and is associated with poor outcome to adjuvant trastuzumab in HER2-positive breast cancer
Source: BMC Cancer. 2017 Dec 28;17:895. doi: 10.1186/s12885-017-3897-x (PMC5745882; doi:10.1186/s12885-017-3897-x)

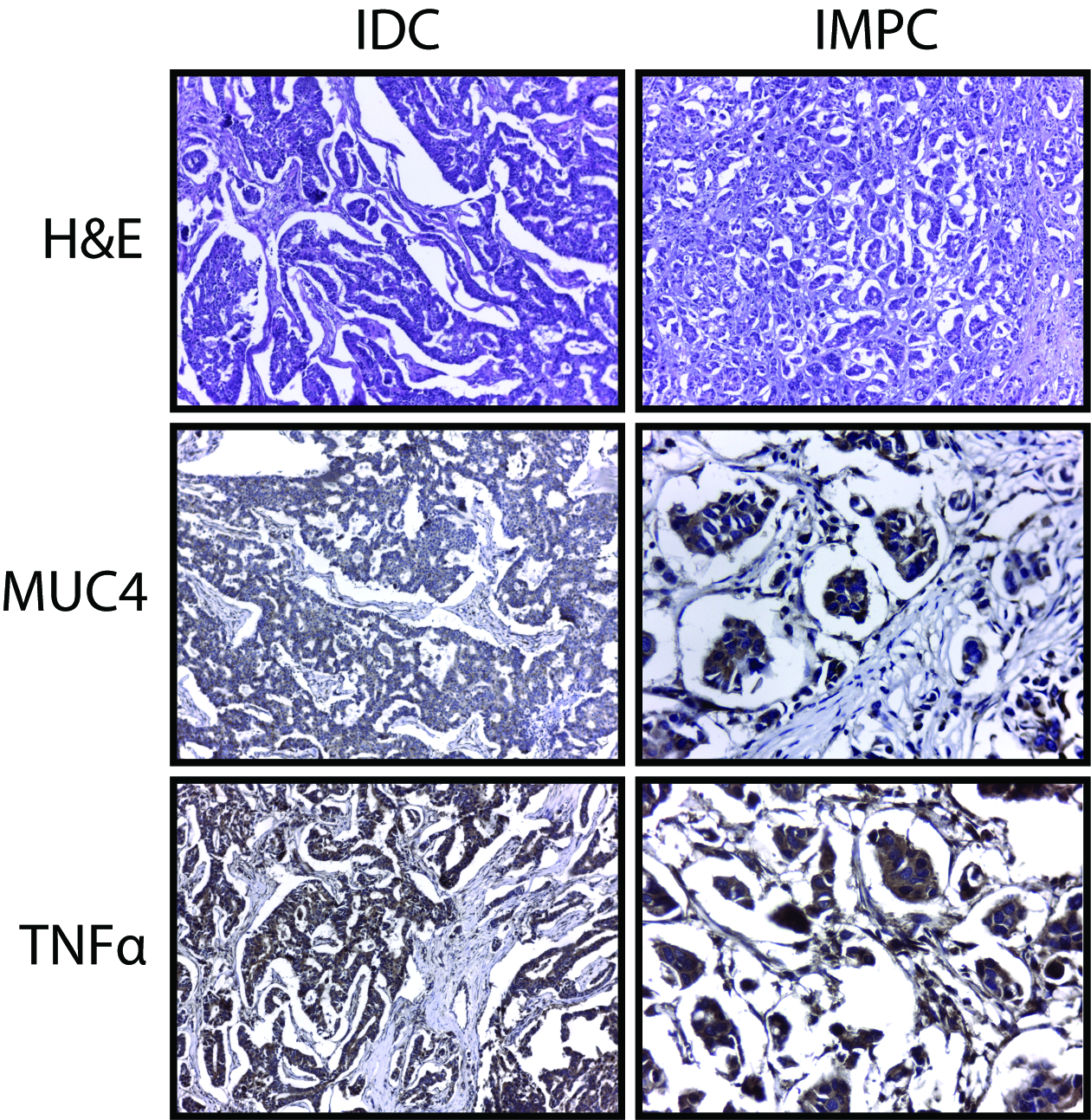

Supplement: Supplementary file 4 — TNFα staining in IDC and IMPC by immunohistochemistry. The panels show representative cases of IDC and IMPC for H&E staining (upper panel), MUC4 (middle panel) and TNFα (lower panel). (TIFF 5225 kb) [file 12885_2017_3897_MOESM4_ESM.tif]
